# Hardy–Weinberg Equilibrium Filtering in Population Genomics: Empirical Review and Decision Framework for Improved Practice

**DOI:** 10.1002/ece3.72688

**Published:** 2026-01-09

**Authors:** Yu‐Hsun Hsu

**Affiliations:** ^1^ Department of Life Sciences National Cheng Kung University Tainan Taiwan

**Keywords:** Hardy–Weinberg, population genomics, population structure, SNP filtering

## Abstract

Hardy–Weinberg equilibrium (HWE) filtering remains widely used in population genomics, but its application remains inconsistent, often lacking detailed justification, and not always aligned with biological context. To evaluate whether conceptual awareness has translated into methodological change, we review empirical studies citing Pearman et al. (2022), a representative study testing the impacts of different grouping approaches for HWE filtering. While pooled filtering is becoming rare, we found a decreasing but still considerable heterogeneity in the decision of filtering schemes, limited reporting of thresholds, and few explicit justifications for applied approaches. These patterns suggest that awareness of HWE filtering limitations is increasing but has not yet led to consistent practice. We synthesise the biological and technical causes of HWE deviation, review recent advances, including population‐aware and structure‐informed filtering tools, and propose a transparent decision framework for population genomic studies. Rather than a default quality‐control step, HWE filtering should be applied as a hypothesis‐aware decision that reflects study aims and biological context. A citation‐based mini‐survey and decision workflow are provided to support biologically informed and reproducible applications.

## Graphical abstract

Hardy–Weinberg equilibrium (HWE) filtering is widely used in population genomics, but its application remains inconsistent and often lacks biological justification. This review went through recent empirical studies that demonstrate awareness of these issues to assess whether conceptual understanding has led to improved practice. This review synthesises the causes of HWE deviation and proposes a biologically informed decision workflow to support transparent filtering applications.

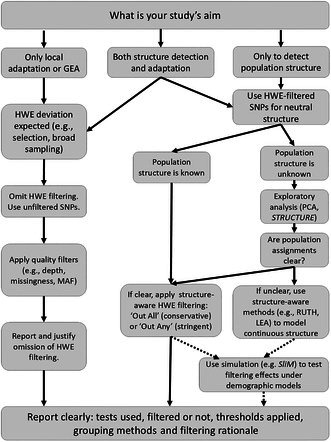

## 1 Introduction

Identifying adaptive units is central to understanding how species respond to environmental change and informing conservation strategies (Faske et al. 2021; Yang et al. 2025). SNP (single‐nucleotide polymorphism)‐based analyses, either through reduced‐representation sequencing methods (e.g., RADseq, restriction site‐associated DNA sequencing (Davey and Blaxter 2010)) or whole genome methods (e.g., whole‐genome sequencing (Li et al. 2009)), have enabled fine‐scale analyses of genome‐wide variation in non‐model taxa to detect cryptic structure and local adaptation in wild populations through increasingly standardised analytical pipelines. Now widely used because they provide high marker density at moderate cost, these approaches have also increased dataset complexity, highlighting the need to revisit assumptions embedded in population genomic filtering approaches.

Among these filtering assumptions, the Hardy–Weinberg Equilibrium (HWE) test provides a null expectation of genotype frequencies under random mating, absence of selection, migration, and mutation, and with infinite population size (Hardy 1908; Weinberg 1908). The HWE test has been used to identify loci potentially affected by selection or genotyping artefacts, especially in earlier studies based on microsatellites (Balloux and Lugon‐Moulin 2002; Hsu et al. 2010; Selkoe and Toonen 2006). With the rise of whole‐genome SNP‐based datasets, HWE filtering has been repurposed as a pre‐processing step to exclude unreliable loci, alongside other filters through the commonly applied analytical pipelines (Linck and Battey 2019; O'Leary et al. 2018; Purcell et al. 2007). However, applying this filter without considering population structure or technical variation risks introducing circularity, obscuring biologically meaningful signals, and reducing the power to detect structure or adaptation (Pearman et al. 2022).

These concerns are especially relevant in wildlife and conservation genomics, where population structure is often unknown and central to the research question itself (Cerezo et al. 2020; Faske et al. 2021; Wariss et al. 2025; Yang et al. 2025). Despite increasing awareness that pooled HWE filtering can remove loci informative for structure (Pearman et al. 2022; Wahlund 1928), default implementations remain widespread, and guidance on when and how to apply HWE filtering remains inconsistent and under‐discussed.

In this mini‐review, we synthesise recent applications of HWE filtering in the context of population genomics. We revisit the biological and technical causes of HWE deviation, evaluate the consequences of common filtering strategies, and assess whether recent studies have adopted population‐aware approaches. Drawing on a citation‐based survey for studies aware of the limitation of HWE filtering (i.e., studies citing Pearman et al. 2022; see Section 4) and conceptual synthesis, we clarify when and how HWE filtering is recommended to be applied, and offer practical guidelines for making biologically informed, statistically robust decisions in diverse research contexts. This review applies primarily to SNP‐based approaches, such as RADseq, double‐digest RADseq (ddRADseq) (Peterson et al. 2012), genotype‐by‐sequencing (GBS) (Elshire et al. 2011), diversity array technology sequencing (DArTseq) (Jaccoud et al. 2001), and low‐coverage whole‐genome sequencing (lcWGS) (Converge consortium 2015; Lou et al. 2021), where SNP filtering is particularly relevant given the large number of loci and variable coverage. Nevertheless, the filtering principles and interpretive framework also extend to other marker systems, such as microsatellites.

## 2 Why Loci Deviate From HWE

Understanding the causes of deviation from HWE is the first step to evaluating whether a filtering approach is appropriate. Deviation from HWE can arise through both biological processes and technical artefacts, and distinguishing between them is essential for population genomic studies. On the one hand, because HWE assumes random mating, no selection, migration, or mutation, and an infinitely large population (Hardy 1908; Weinberg 1908), deviations from it can arise from a range of biologically meaningful processes. Inbreeding and assortative mating are two forms of non‐random mating known to reduce heterozygosity (Kardos et al. 2016). Balancing selection, large effective population size, and clonal reproduction may lead to heterozygote excess (Abramovs et al. 2020; Neamatzadeh et al. 2024). Alternatively, in structured populations, allele frequencies differ among subgroups, so pooling them will result in heterozygote deficiency, a phenomenon known as the Wahlund effect (Wahlund 1928). Although sometimes conflated with genotyping artefacts, this is a predictable consequence of population structure and should not be treated as noise.

On the other hand, technical artefacts such as scoring errors can also cause apparent deviations. For example, in RADseq datasets, allele dropout due to mutations near restriction sites may result in null alleles, while PCR duplicates and uneven coverage can distort allele frequencies (Andrews et al. 2016; Bresadola et al. 2020). These technical errors can mimic biological signals, inflate estimates such as *F*_ST_ and effective population size, and bias downstream analyses (Bresadola et al. 2020; Marandel et al. 2020; Shafer et al. 2017).

However, these two main sources of HWE deviation often confound with one another and are hard to distinguish (Bresadola et al. 2020; Díaz‐Arce and Rodríguez‐Ezpeleta 2019). This is particularly problematic in studies lacking prior knowledge of structure, where filtering may inadvertently remove loci carrying an informative signal (Pearman et al. 2022). Therefore, decisions regarding HWE filtering should be made with attention to both the biological context and data‐specific artefacts. To explore how these concerns are reflected in recent practice and the recent difficulties, we next review recent trends of HWE filtering in wildlife population studies.

## 3 Recent Trends and Difficulties of HWE Filtering in Wildlife Population Structure Studies

Originally intended to remove unreliable loci, HWE filtering is still frequently applied across pooled samples even when population structure is unknown. This pooled filtering approach, referred to as “Out Combo” by Pearman et al. (2022), removes loci deviating from HWE across pooled samples and is implemented as the default in widely used tools such as VCFtools and STACKS (Danecek et al. 2011; Rochette et al. 2019). However, analysing pooled samples masks allele‐frequency differences among populations. As a result, the Out Combo scheme may remove loci whose allele frequency differences reflect population structure, thereby reducing clustering resolution and biasing downstream analyses (Pearman et al. 2022).

Alternative filtering schemes include “Out Any”, which excludes loci deviating from HWE in any population, “Out Within”, which excludes loci only from populations in which they deviate from HWE, or “Out All”, which removes loci only if they deviate in all populations (Pearman et al. 2022). According to both simulated and empirical data, these filtering schemes offer greater biological realism because they reflect population boundaries, but each comes with trade‐offs. The “Out Any” and “Out Within” options are more stringent and may remove loci under local adaptation, whereas the “Out All” option is more conservative and retains structure‐information loci. Therefore, neither of these schemes is universally preferable and may require prior knowledge or hypotheses about population structure.

Recent studies illustrate a wide range of HWE filtering practices, differing in when filtering is applied and which schemes are used. For example, after structure inference, an Out Any HWE filtering scheme was applied across defined populations in a study of invasive common mynas ( *Acridotheres tristis* ) (Atsawawaranunt et al. 2023). A population‐aware HWE filtering scheme has also been applied in studies of seagrass ( *Zostera marina* ), notothenioid fish ( *Harpagifer antarcticus* ), and bank vole ( *Clethrionomys glareolus* ) (Bernal‐Durán et al. 2024; Markova et al. 2023; Ortiz et al. 2025). Alternatively, de Greef et al. (2022) evaluated the estimation of population structure with and without HWE filtering in their study of the northern bottlenose whale ( *Hyperoodon ampullatus* ), noting minimal differences but advocating for sensitivity analyses.

Variations in HWE tests also come from the fact that some studies did not provide critical filtering details, such as whether HWE filtering was performed, which filtering scheme was fitted, or what thresholds and multiple‐test corrections were applied (Cano et al. 2022; Orita et al. 2021; Sethuraman et al. 2019; Wariss et al. 2025; Yang et al. 2025). Reporting such thresholds and multiple‐testing corrections for HWE filtering is crucial for transparency and comparability across studies (Hemstrom et al. 2024; Sethuraman et al. 2019). These omissions may be caused by uncertainty about population structure, limited resolution in sampling, or assumptions of panmixia (Atsawawaranunt et al. 2023; Cano et al. 2022; Lin et al. 2024; Wariss et al. 2025; Yang et al. 2025), but are indistinguishable in cases without explicit reporting.

To evaluate how conceptual concerns have been incorporated into recent empirical practice, particularly among researchers likely to be aware of these issues, we next review recent studies that cite Pearman et al. (2022), a representative reference that systematically defined and compared HWE filtering schemes and evaluated their effects with both empirical and simulated data.

## 4 Mini‐Survey: Are Population‐Aware Practices Becoming Standard?

Despite growing recognition that HWE filtering can bias population genomic inferences if applied indiscriminately, it remains unclear whether empirical studies have shifted toward more nuanced, population‐aware practices. To assess whether conceptual advances have led to practical changes in filtering strategies, we conducted a focused mini‐survey of empirical studies citing Pearman et al. (2022). Our goal was to assess how many of these citing studies have adopted population‐aware filtering protocols, explicitly justified their use or non‐use of HWE filters, and reported their filtering strategies transparently.

**FIGURE 1:**
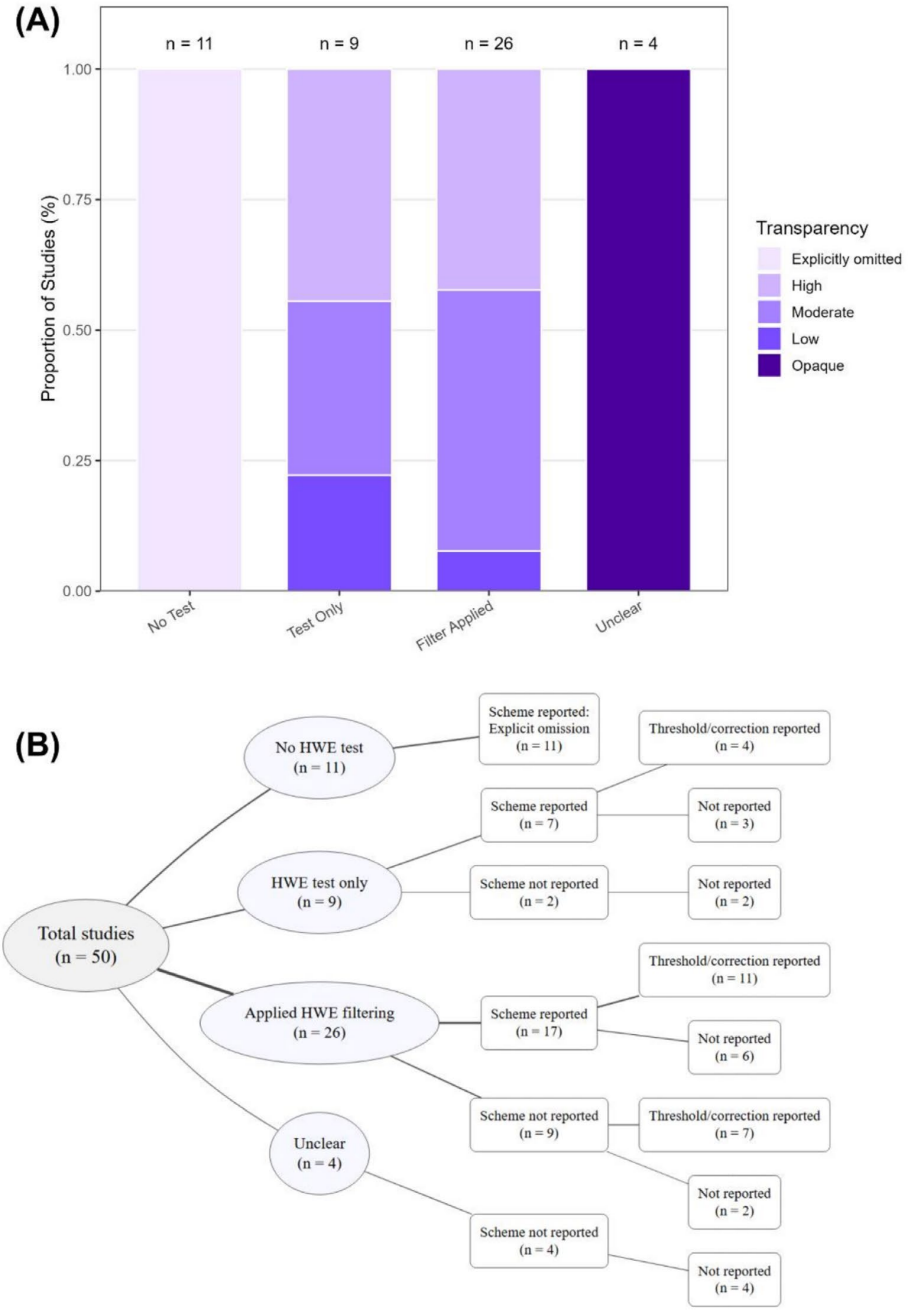
Summary of HWE filtering practices in 50 empirical studies that cited Pearman et al. (2022). (A) Proportion of studies grouped by whether they tested for HWE, applied HWE filtering, and their corresponding transparency levels (see Methods for definitions). (B) Decision tree illustrating how these studies were distributed based on whether they tested for HWE, whether they reported the filtering scheme used, and whether they reported threshold values or multiple‐test correction procedures. Node labels give the number of studies in each category; line width is proportional to the number of studies following each branch, with thicker lines indicating branches containing more studies.

A total of 70 citing articles were retrieved via Google Scholar on 2 July 2025. After excluding non‐empirical studies (six reviews and five methodological papers), duplicates (three preprints and three archived theses), and inaccessible articles (*n* = 3), 50 studies remained for assessment (Figure 1). For each study, we recorded whether they applied HWE filtering or HWE testing, their specific filtering/testing scheme, the threshold value used, and the methods employed for multiple‐test correction (Table S1). These studies represent a group that, by citing Pearman et al. (2022), can reasonably be assumed to have been aware of the weakness of HWE filtering in structured populations.

To summarise reporting quality across studies, we evaluated three criteria of HWE filtering transparency: (1) explicitly reporting whether they applied the HWE test only or HWE filtering, and acknowledged the risk of removing informative SNPs; (2) categorising or describing the HWE filtering scheme; and (3) providing a clear filtering threshold and/or multiple‐testing correction. Based on these criteria, we categorised studies into four levels of transparency: *high*—all criteria reported; *moderate*—criterion (1) plus one additional criterion met; *low*—only criterion (1) met; and *opaque*—criterion (1) not reported. In addition, a few studies explicitly stated that they omitted HWE filtering to avoid potential biases. These studies were classified as “explicitly omitted” to reflect their transparent justification for not applying HWE filtering.

Based on these criteria, 15 were classified as having “high” transparency, of which 11 applied HWE filtering and four did not (Figure 1 and Table S1). Together with the 11 studies that explicitly omitted the HWE test and 16 studies scored as “moderate”, this means 84% of the reviewed studies demonstrated moderate to high awareness of the potential impacts of HWE in their empirical studies. Among the eight studies scored as “low” or “opaque”, two performed HWE tests without filtering, so the absense of detailed information on their test scheme or threshold had minimal or no effect on their results. On the contrary, four studies did not specify whether they applied the HWE test or filtering, despite citing Pearman et al. (2022), raising concerns about procedural transparency.

Among these 50 reviewed studies, 20 studies (40%) explicitly stated that they omitted the HWE test or filtering and provided reasoning, most commonly to avoid removing informative loci or due to biological expectations of natural deviation from equilibrium (Figure 1 and Table S1). For example, Aleman, Dorken, et al. (2024) and Grant et al. (2022) stated broad geographic sampling and heterogeneous selection regimes as reasons to avoid HWE‐based exclusion. Among the 35 studies that applied “Test Only” HWE test or HWE filtering, filtering schemes varied widely, spanning all four filtering schemes tested and discussed in Pearman et al. (2022). Notably, 21 reviewed studies (42%) applied a structure‐aware filtering scheme (“Out All”, “Out Any”, or “Out Within”), showing an increase from 10.1% in Pearman et al. (2022) to 42% after excluding studies applying the potentially problematic “Out Combo” and “Out Some”.

In addition to formal HWE tests, some studies in our survey applied heterozygosity (He)‐based thresholds to identify loci with unusually high He (de Greef et al. 2022; de Silva 2023; Holt et al. 2023). Although not a formal HWE test, this approach removes loci deviating from HWE, often corresponding to paralogous or duplicated loci (McKinney et al. 2017), and is therefore considered alongside HWE filtering approaches in this review.

Overall, this survey reveals a mixed but positive pattern of awareness of HWE filtering: while heterogeneity in filtering schemes persists, there is growing transparency in the implementation of HWE testing (Figure 1). Therefore, population‐aware HWE filtering practices are emerging, although they are not yet standardised. These trends suggest that conceptual awareness of HWE filtering is beginning to translate into more consistent and reproducible analytical practices.

In the next section, we review potential solutions drawn from structured systems and model‐based approaches that may offer more robust alternatives for wildlife genomics.

## 5 Methodological Alternatives to Conventional HWE Filtering

In response to the limitations of pooled HWE filtering, several biologically informed and statistically robust alternatives have emerged, from population‐aware filtering, ancestry‐informed HWE tests, to simulation‐based and machine‐learning approaches. These approaches are particularly important for studies aiming to infer subtle population structure, detect signals of local adaptation, or conduct genotype–environment association (GEA) analyses, where inappropriate filtering may bias or obscure the desired signal. Population‐aware filtering schemes such as “Out All” retain informative variation while minimising bias. Nevertheless, such a filtering scheme should be applied after structure inference (e.g., via PCA, ADMIXTURE) to avoid circularity. To enable evaluation of alternative strategies, Armstrong et al. (2025) developed a modular pipeline with HWE filtering as one adjustable component. This pipeline enables users to explore how different filtering options affect locus retention and the downstream inference of population structure.

When population assignments are unclear, or admixture is expected, ancestry‐informed tests such as RUTH (Robust Unified Test for HWE) (Kwong et al. 2021) offer a useful alternative. RUTH integrates genotype likelihoods and individual‐level ancestry into HWE tests, improving robustness in low‐coverage or admixed datasets by directly modeling latent structure. In contrast, model‐based clustering approaches, such as entropy (Gompert et al. 2014) or LEA (Frichot and François 2015), do not assume HWE a priori. Instead, they infer population structure from genotype data while allowing HWE deviations to emerge as part of the output. These methods are especially useful when the structure is weak or continuous.

Simulations provide approaches to evaluate the consequences of filtering by generating synthetic data under specified demographic scenarios. For example, *SLiM* applied simulations incorporating selection, non‐random mating, migration, and changes in population size to test the effects of demography or selection (Haller and Messer 2019). Similarly, *fastsimcoal2* performs coalescent‐based simulations of SNP datasets under various demographic models, including structure, bottlenecks, and gene flow (Excoffier et al. 2021). Both methods generate simulated datasets with known parameters to evaluate the accuracy of filtering schemes. These simulations provide a framework for assessing filtering effects, and researchers can then evaluate how different HWE filters affect *F*_ST_, clustering, or the detection of outlier loci.

In addition, the recent emergence of machine learning approaches also offers promising alternatives to rule‐based SNP filtering. Instead of applying fixed thresholds for filtering, these methods apply statistical learning algorithms to evaluate multiple variant‐level features simultaneously. For example, *ForestQC* (Li et al. 2019) applies a random forest classifier to distinguish high‐ from low‐quality SNPs using a combination of indicators. Similarly, *DeepVariant* (Poplin et al. 2018) applies a deep neural network to predict genotype likelihoods directly from raw sequencing data, integrating diverse signals to assess variant confidence.

Beyond HWE, population structure similarly influences other filtering criteria, such as linkage disequilibrium (LD), where uncorrected structure can inflate LD estimates and lead to excessive marker pruning. Recent studies have developed structure‐aware LD filtering methods to address this issue (Bercovich et al. 2025). These parallels show a broader need for filtering frameworks that consider demographic context and population stratification, ensuring that quality control enhances rather than obscures the biological signal of interest.

## 6 Practical Workflow for Population‐Structure–Aware HWE Filtering

Based on the methodological developments and alternative solutions reviewed in earlier sections, we present a set of practical recommendations for whether or how to apply HWE filtering in population genomic studies. These recommendations are context‐dependent and relate to study aims and the expected degree of population structure, particularly those involving non‐model organisms and reduced‐representation sequencing.

First, HWE should be applied in accordance with study objectives and biological context. For example, HWE filtering is often unnecessary and potentially misleading in studies focused solely on local adaptation, genotype–environment associations (GEA), or other scenarios where HWE deviation is biologically expected (Aleman, Arteaga, et al. 2024; Aleman, Dorken, et al. 2024; de Greef et al. 2022; Ellis et al. 2023; Ellis et al. 2024; Muharromah et al. 2024) to avoid biasing the results. Alternatively, these studies are suggested to rely on other standard quality filters, such as read depth, missingness, or minor allele frequency (MAF), to remove loci with artefacts (Linck and Battey 2019; O'Leary et al. 2018; Purcell et al. 2007).

Second, HWE filtering can be applied in studies aiming to infer population structure or investigate neutral population processes, but only after population structure has been assessed. Structure‐assess analyses (e.g., PCA, STRUCTURE) can be performed first to infer the population structure, followed by population‐aware HWE filtering. Among the available filtering schemes, “Out All” offers a conservative option that retains loci with structure‐related or context‐dependent deviations. In contrast, “Out Any” is more stringent and may remove loci under local adaptation or drift. Additionally, a sensitivity analysis that compares multiple filtering schemes can be applied to identify the effects of filtering on the results.

Third, for datasets with weak population structure or subtle population boundaries, structure‐aware tests such as RUTH (Kwong et al. 2021) will be helpful. Alternatively, clustering methods that do not assume HWE, such as entropy and *LEA* (Frichot and François 2015; Gompert et al. 2014), can infer structure and genotype likelihoods without biasing results through pre‐filtering. Simulation‐based methodologies, like *SLiM* and *fastsimcoal2*, can further assist in evaluating how HWE filtering affects downstream analyses under complex demographic scenarios (Excoffier et al. 2021; Haller and Messer 2019).

**FIGURE 2:**
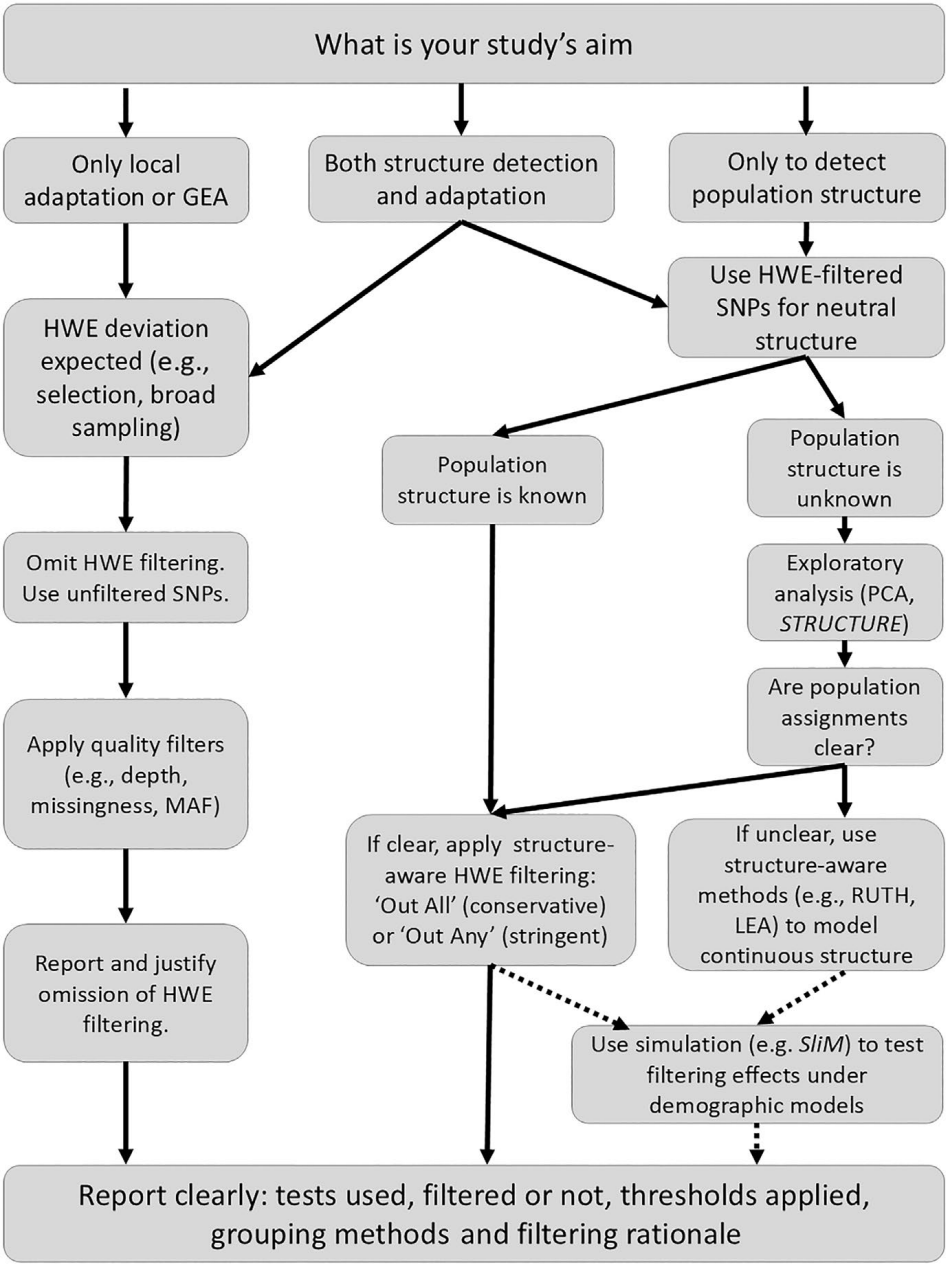
Decision workflow for applying HWE filtering in population genomic studies. This flowchart provides a flexible guide to determine whether, when, and how to apply HWE filtering, based on study goals (e.g., local adaptation, genotype–environment association (GEA), or population structure inference) and data characteristics (e.g., known vs. unknown structure). Rather than a rigid protocol, this framework encourages biologically informed and reproducible choices tailored to each study's design and objectives. Solid lines indicate recommended steps; dotted lines indicate optional steps (MAF, minor allele frequency; other terms are defined in the main text).

Finally, transparent reporting remains essential. It is recommended to explicitly report whether HWE filtering was used, the specific tests and thresholds applied, whether multiple‐testing corrections were implemented, and how the populations were defined. For studies that include both population structure inference and downstream analyses such as GEA or selection scans, HWE filtering can be applied to assist structure detection, but the filtered dataset is not suggested to be reused for downstream adaptation analyses. Therefore, it is recommended to use unfiltered SNPs for GEA or adaptation detection analyses to avoid inadvertently removing loci under selection. This double approach helps reduce false positives in structure inference while preserving adaptive signals. A summary decision workflow is provided in Figure 2.

## 7 Conclusion

Despite increasing awareness of the limitations of HWE filtering (Pearman et al. 2022), this widely used filter for SNP loci remains inconsistently applied in population genomics. In this mini‐review, we surveyed recent empirical studies that cited Pearman et al. (2022) and found that approximately 40% of the studies either explicitly omitted HWE filtering or conducted HWE for test only. Among those applying filtering, the proportion using structure‐aware approaches increased from 10.1% (Pearman et al. 2022) to 42% in our survey, after excluding studies that used potentially problematic “Out Combo” or “Out Some” schemes. Notably, > 80% of the reviewed studies reported at least one testing/filtering detail, such as filtering scheme, threshold, or multiple test correction, even if not all components were always provided (Table S1 and Figure 1). This pattern suggests a shift from directly applying the default HWE filtering setting toward a more context‐dependent, biologically informed filtering decision.

While this survey is restricted to studies citing Pearman et al. (2022) to ensure a comparable conceptual baseline, exploring broader community‐wide trends may be possible in future studies using different conceptual frameworks or methodological approaches. Meanwhile, to enhance this development, we proposed a workflow to apply HWE filtering that should be reframed as a biologically informed decision, guided by study aims, population structure, and the interpretive value of HWE deviation (Figure 2). When used thoughtfully, HWE can serve as a hypothesis‐generating tool to reveal signals of structure, selection, or mating patterns rather than obscuring them. As genomic studies move toward more flexible, system‐specific frameworks, standardising filtering logic and improving reporting practices will be essential for robust and reproducible research across ecological and evolutionary contexts.

## Author Contributions

**Yu‐Hsun Hsu:** conceptualization (lead), data curation (lead), funding acquisition (lead), investigation (lead), visualization (lead), writing – original draft (lead), writing – review and editing (lead).

## Funding

This project is supported by Research Grant NSTC 114‐2621‐B‐006‐006 from the National Science and Technology Council (NSTC), Taiwan, to YHH.

## Conflicts of Interest

The author declares no conflicts of interest.

## Supporting information


**Table S1:** This table summarises the 50 studies in our analysis after screening articles citing Pearman et al. 2022. The 20 additional entries were excluded due to article type, duplication, or inaccessibility (see Section 4 for details).

## Data Availability

All data used in this study have already been provided in the content.

## References

[ece372688-bib-0001] Abramovs, N. , A. Brass , and M. Tassabehji . 2020. “Hardy‐Weinberg Equilibrium in the Large Scale Genomic Sequencing Era.” Frontiers in Genetics 11: 210. 10.3389/fgene.2020.00210. 32231685 PMC7083100

[ece372688-bib-0002] Aleman, A. , M. C. Arteaga , J. Gasca‐Pineda , and R. Bello‐Bedoy . 2024. “Divergent Lineages in a Young Species: The Case of Datilillo (*Yucca Valida*), a Broadly Distributed Plant From the Baja California Peninsula.” American Journal of Botany 111, no. 9: e16385. 10.1002/ajb2.16385. 39113241

[ece372688-bib-0003] Aleman, A. , M. E. Dorken , A. B. A. Shafer , T. Patel , P. A. Volkova , and J. R. Freeland . 2024. “Development of Genomic Resources for Cattails (*Typha*), a Globally Important Macrophyte Genus.” Freshwater Biology 69, no. 1: 74–83. 10.1111/fwb.14194.

[ece372688-bib-0004] Andrews, K. R. , J. M. Good , M. R. Miller , G. Luikart , and P. A. Hohenlohe . 2016. “Harnessing the Power of RADseq for Ecological and Evolutionary Genomics.” Nature Reviews Genetics 17, no. 2: 81–92. 10.1038/nrg.2015.28. PMC4823021 26729255

[ece372688-bib-0005] Armstrong, E. E. , C. Y. Li , M. G. Campana , et al. 2025. “A Pipeline and Recommendations for Population and Individual Diagnostic SNP Selection in Non‐Model Species.” Molecular Ecology Resources 25, no. 3: e14048. 10.1111/1755-0998.14048. 39611246 PMC11887608

[ece372688-bib-0006] Atsawawaranunt, K. , K. M. Ewart , R. E. Major , R. N. Johnson , A. W. Santure , and A. Whibley . 2023. “Tracing the Introduction of the Invasive Common Myna Using Population Genomics.” Heredity 131, no. 1: 56–67. 10.1038/s41437-023-00621-w. 37193854 PMC10313710

[ece372688-bib-0007] Balloux, F. , and N. Lugon‐Moulin . 2002. “The Estimation of Population Differentiation With Microsatellite Markers.” Molecular Ecology 11, no. 2: 155–165. 10.1046/j.0962-1083.2001.01436.x. 11856418

[ece372688-bib-0008] Bercovich, U. , M. S. Rasmussen , Z. Li , C. Wiuf , and A. Albrechtsen . 2025. “Measuring Linkage Disequilibrium and Improvement of Pruning and Clumping in Structured Populations.” Genetics 229, no. 3: iyaf009. 10.1093/genetics/iyaf009. 39907701

[ece372688-bib-0009] Bernal‐Durán, V. , D. Donoso , A. Pinones , et al. 2024. “Combining Population Genomics and Biophysical Modelling to Assess Connectivity Patterns in an Antarctic Fish.” Molecular Ecology 33, no. 11: e17360. 10.1111/mec.17360. 38656687

[ece372688-bib-0010] Bresadola, L. , V. Link , C. A. Buerkle , C. Lexer , and D. Wegmann . 2020. “Estimating and Accounting for Genotyping Errors in RAD‐Seq Experiments.” Molecular Ecology Resources 20, no. 4: 856–870. 10.1111/1755-0998.13153. 32142201

[ece372688-bib-0011] Cano, M. J. , A. D. Twyford , and P. M. Hollingsworth . 2022. “Population and Conservation Genetics Using RAD Sequencing in Four Endemic Conifers From South America.” Biodiversity and Conservation 31, no. 13–14: 3093–3112. 10.1007/s10531-022-02471-0.

[ece372688-bib-0012] Cerezo, M. L. M. , M. Kucka , K. Zub , Y. F. Chan , and J. Bryk . 2020. “Population Structure of *Apodemus flavicollis* and Comparison to *Apodemus sylvaticus* in Northern Poland Based on RAD‐Seq.” BMC Genomics 21, no. 1: 241. 10.1186/s12864-020-6603-3. 32183700 PMC7079423

[ece372688-bib-0013] Converge consortium . 2015. “Sparse Whole‐Genome Sequencing Identifies Two Loci for Major Depressive Disorder.” Nature 523, no. 7562: 588–591. 10.1038/nature14659. 26176920 PMC4522619

[ece372688-bib-0014] Danecek, P. , A. Auton , G. Abecasis , et al. 2011. “The Variant Call Format and VCFtools.” Bioinformatics 27, no. 15: 2156–2158. 10.1093/bioinformatics/btr330. 21653522 PMC3137218

[ece372688-bib-0015] Davey, J. L. , and M. W. Blaxter . 2010. “RADSeq: Next‐Generation Population Genetics.” Briefings in Functional Genomics 9, no. 5–6: 416–423. 10.1093/bfgp/elq031. 21266344 PMC3080771

[ece372688-bib-0016] de Greef, E. , A. L. Einfeldt , P. J. O. Miller , et al. 2022. “Genomics Reveal Population Structure, Evolutionary History, and Signatures of Selection in the Northern Bottlenose Whale, *Hyperoodon ampullatus* .” Molecular Ecology 31, no. 19: 4919–4931. 10.1111/mec.16643. 35947506 PMC9804413

[ece372688-bib-0017] de Silva, N. P. 2023. Using Ecological and Genomic Approaches to Restore Australian Grasslands in the Face of Global Change. Monash University.

[ece372688-bib-0018] Díaz‐Arce, N. , and N. Rodríguez‐Ezpeleta . 2019. “Selecting RAD‐Seq Data Analysis Parameters for Population Genetics: The More the Better?” Frontiers in Genetics 10: 533. 10.3389/fgene.2019.00533. 31191624 PMC6549478

[ece372688-bib-0019] Ellis, C. D. , K. L. MacLeod , T. L. Jenkins , et al. 2023. “Shared and Distinct Patterns of Genetic Structure in Two Sympatric Large Decapods.” Journal of Biogeography 50, no. 7: 1271–1284. 10.1111/jbi.14623.

[ece372688-bib-0020] Ellis, C. D. , J. R. Paris , T. L. Jenkins , et al. 2024. “Genetic Divergence and Adaptation of an Isolated European Lobster Population in The Netherlands.” ICES Journal of Marine Science 81, no. 6: 1039–1052. 10.1093/icesjms/fsae059.

[ece372688-bib-0021] Elshire, R. J. , J. C. Glaubitz , Q. Sun , et al. 2011. “A Robust, Simple Genotyping‐By‐Sequencing (GBS) Approach for High Diversity Species.” PLoS One 6, no. 5: e19379. 10.1371/journal.pone.0019379. 21573248 PMC3087801

[ece372688-bib-0022] Excoffier, L. , N. Marchi , D. A. Marques , R. Matthey‐Doret , A. Gouy , and V. C. Sousa . 2021. “ *fastsimcoal2*: Demographic Inference Under Complex Evolutionary Scenarios.” Bioinformatics 37, no. 24: 4882–4885. 10.1093/bioinformatics/btab468. 34164653 PMC8665742

[ece372688-bib-0023] Faske, T. M. , A. C. Agneray , J. P. Jahner , L. M. Sheta , E. A. Leger , and T. L. Parchman . 2021. “Genomic and Common Garden Approaches Yield Complementary Results for Quantifying Environmental Drivers of Local Adaptation in Rubber Rabbitbrush, a Foundational Great Basin Shrub.” Evolutionary Applications 14, no. 12: 2881–2900. 10.1111/eva.13323. 34950235 PMC8674890

[ece372688-bib-0024] Frichot, E. , and O. François . 2015. “LEA: An R Package for Landscape and Ecological Association Studies.” Methods in Ecology and Evolution 6, no. 8: 925–929. 10.1111/2041-210x.12382.

[ece372688-bib-0025] Gompert, Z. , L. K. Lucas , C. A. Buerkle , M. L. Forister , J. A. Fordyce , and C. C. Nice . 2014. “Admixture and the Organization of Genetic Diversity in a Butterfly Species Complex Revealed Through Common and Rare Genetic Variants.” Molecular Ecology 23, no. 18: 4555–4573. 10.1111/mec.12811. 24866941

[ece372688-bib-0059] Grant, E. H. C. , K. P. Mulder , A. B. Brand , et al. 2022. “Speciation with Gene Flow in a Narrow Endemic West Virginia Cave Salamander (Gyrinophilus subterraneus).” Conservation Genetics 23, no. 4: 727–744. 10.1007/s10592-02201445-7.

[ece372688-bib-0026] Haller, B. C. , and P. W. Messer . 2019. “SLiM 3: Forward Genetic Simulations Beyond the Wright‐Fisher Model.” Molecular Biology and Evolution 36, no. 3: 632–637. 10.1093/molbev/msy228. 30517680 PMC6389312

[ece372688-bib-0027] Hardy, G. H. 1908. “Mendelian Proportions in a Mixed Population.” Yale Journal of Biology and Medicine 76, no. 2: 79–80. 10.1126/science.28.706.49. PMC2582692 15369635

[ece372688-bib-0028] Hemstrom, W. , J. A. Grummer , G. Luikart , and M. R. Christie . 2024. “Next‐Generation Data Filtering in the Genomics Era.” Nature Reviews Genetics 25, no. 11: 750–767. 10.1038/s41576-024-00738-6. 38877133

[ece372688-bib-0029] Holt, J. R. , J. M. Lerma , T. J. Raszick , and R. F. Medina . 2023. “High‐Resolution Population Genetic Structure of Tawny Crazy Ant (*Nylanderia fulva Mayr*: Hymenoptera: Formicidae) From the Origin in South America and Introduced Regions of the United States.” 10.21203/rs.3.rs-2399319/v1.

[ece372688-bib-0030] Hsu, Y. H. , L. L. Severinghaus , S. H. Li , and Y. C. Hsu . 2010. “Detecting Obscure Hybrids of Light‐Vented Bulbul (*Pycnonotus Sinensis Formosae*) and Styan's Bulbul (*P. Taivanus*) With Microsatellite.” Journal of National Park 20, no. 1: 26–36.

[ece372688-bib-0031] Jaccoud, D. , K. Peng , D. Feinstein , and A. Kilian . 2001. “Diversity Arrays: A Solid State Technology for Sequence Information Independent Genotyping.” Nucleic Acids Research 29, no. 4: E25. 10.1093/nar/29.4.e25. 11160945 PMC29632

[ece372688-bib-0032] Kardos, M. , H. R. Taylor , H. Ellegren , G. Luikart , and F. W. Allendorf . 2016. “Genomics Advances the Study of Inbreeding Depression in the Wild.” Evolutionary Applications 9, no. 10: 1205–1218. 10.1111/eva.12414. 27877200 PMC5108213

[ece372688-bib-0033] Kwong, A. M. , T. W. Blackwell , J. LeFaive , et al. 2021. “Robust, Flexible, and Scalable Tests for Hardy‐Weinberg Equilibrium Across Diverse Ancestries.” Genetics 218, no. 1: iyab044. 10.1093/genetics/iyab044. 33720349 PMC8128395

[ece372688-bib-0034] Li, J. , B. Jew , L. Zhan , et al. 2019. “ForestQC: Quality Control on Genetic Variants From Next‐Generation Sequencing Data Using Random Forest.” PLoS Computational Biology 15, no. 12: e1007556. 10.1371/journal.pcbi.1007556. 31851693 PMC6938691

[ece372688-bib-0035] Li, R. , Y. Li , X. Fang , et al. 2009. “SNP Detection for Massively Parallel Whole‐Genome Resequencing.” Genome Research 19, no. 6: 1124–1132. 10.1101/gr.088013.108. 19420381 PMC2694485

[ece372688-bib-0036] Lin, X. N. , C. Y. Ma , L. S. Hu , et al. 2024. “Genomics‐Informed Range Predictions Under Global Warming Reveal Reduced Adaptive Diversity Whilst Buffering Range Shifts for a Marine Snail.” Global Change Biology 30, no. 11: e17571. 10.1111/gcb.17571. 39523661

[ece372688-bib-0037] Linck, E. , and C. J. Battey . 2019. “Minor Allele Frequency Thresholds Strongly Affect Population Structure Inference With Genomic Data Sets.” Molecular Ecology Resources 19, no. 3: 639–647. 10.1111/1755-0998.12995. 30659755

[ece372688-bib-0038] Lou, R. N. , A. Jacobs , A. P. Wilder , and N. O. Therkildsen . 2021. “A Beginner's Guide to Low‐Coverage Whole Genome Sequencing for Population Genomics.” Molecular Ecology 30, no. 23: 5966–5993. 10.1111/mec.16077. 34250668

[ece372688-bib-0039] Marandel, F. , G. Charrier , J. B. Lamy , S. Le Cam , P. Lorance , and V. M. Trenkel . 2020. “Estimating Effective Population Size Using RADseq: Effects of SNP Selection and Sample Size.” Ecology and Evolution 10, no. 4: 1929–1937. 10.1002/ece3.6016. 32128126 PMC7042749

[ece372688-bib-0040] Markova, S. , H. C. Lanier , M. A. Escalante , et al. 2023. “Local Adaptation and Future Climate Vulnerability in a Wild Rodent.” Nature Communications 14, no. 1: 7840. 10.1038/s41467-023-43383-z. PMC10686993 38030627

[ece372688-bib-0041] McKinney, G. J. , R. K. Waples , L. W. Seeb , and J. E. Seeb . 2017. “Paralogs Are Revealed by Proportion of Heterozygotes and Deviations in Read Ratios in Genotyping‐By‐Sequencing Data From Natural Populations.” Molecular Ecology Resources 17, no. 4: 656–669. 10.1111/1755-0998.12613. 27762098

[ece372688-bib-0042] Muharromah, A. F. , T. M. Carvajal , M. A. F. Regilme , and K. Watanabe . 2024. “Fine‐Scale Adaptive Divergence and Population Genetic Structure of *Aedes Aegypti* in Metropolitan Manila.” Parasites & Vectors 17, no. 1: 233. 10.1186/s13071-024-06300-x. 38769579 PMC11107013

[ece372688-bib-0043] Neamatzadeh, H. , S. A. Dastgheib , M. Mazaheri , et al. 2024. “Hardy‐Weinberg Equilibrium in Meta‐Analysis Studies and Large‐Scale Genomic Sequencing Era.” Asian Pacific Journal of Cancer Prevention 25, no. 7: 2229–2235. 10.31557/APJCP.2024.25.7.2229. 39068553 PMC11480592

[ece372688-bib-0044] O'Leary, S. J. , J. B. Puritz , S. C. Willis , C. M. Hollenbeck , and D. S. Portnoy . 2018. “These Aren't the Loci You're Looking for: Principles of Effective SNP Filtering for Molecular Ecologists.” Molecular Ecology 27, no. 16: 3193–3206. 10.1111/mec.14792. 29987880

[ece372688-bib-0045] Orita, R. , Y. Nagano , Y. Kawamura , K. Kimura , and G. Kobayashi . 2021. “Genetic Diversity and Population Structure of Razor Clam *Sinonovacula Constricta* in Ariake Bay, Japan, Revealed Using RAD‐Seq SNP Markers.” Scientific Reports 11, no. 1: 7761. 10.1038/s41598-021-87395-5. 33833337 PMC8032755

[ece372688-bib-0046] Ortiz, B. A. B. , F. C. Boardman , J. L. Ruesink , and K. A. Naish . 2025. “Adaptive Genetic Differentiation Between Spatially Proximate Annual and Perennial Life History Types of a Marine Foundation Species.” Molecular Ecology 34, no. 8: e17730. 10.1111/mec.17730. 40109014

[ece372688-bib-0047] Pearman, W. S. , L. Urban , and A. Alexander . 2022. “Commonly Used Hardy‐Weinberg Equilibrium Filtering Schemes Impact Population Structure Inferences Using RADseq Data.” Molecular Ecology Resources 22, no. 7: 2599–2613. 10.1111/1755-0998.13646. 35593534 PMC9541430

[ece372688-bib-0048] Peterson, B. K. , J. N. Weber , E. H. Kay , H. S. Fisher , and H. E. Hoekstra . 2012. “Double Digest RADseq: An Inexpensive Method for De Novo SNP Discovery and Genotyping in Model and Non‐Model Species.” PLoS One 7, no. 5: e37135. 10.1371/journal.pone.0037135. 22675423 PMC3365034

[ece372688-bib-0049] Poplin, R. , P. C. Chang , D. Alexander , et al. 2018. “A Universal SNP and Small‐Indel Variant Caller Using Deep Neural Networks.” Nature Biotechnology 36, no. 10: 983–987. 10.1038/nbt.4235. 30247488

[ece372688-bib-0050] Purcell, S. , B. Neale , K. Todd‐Brown , et al. 2007. “PLINK: A Tool Set for Whole‐Genome Association and Population‐Based Linkage Analyses.” American Journal of Human Genetics 81, no. 3: 559–575. 10.1086/519795. 17701901 PMC1950838

[ece372688-bib-0051] Rochette, N. C. , A. G. Rivera‐Colón , and J. M. Catchen . 2019. “Stacks 2: Analytical Methods for Paired‐End Sequencing Improve RADseq‐Based Population Genomics.” Molecular Ecology 28, no. 21: 4737–4754. 10.1111/mec.15253. 31550391

[ece372688-bib-0052] Selkoe, K. A. , and R. J. Toonen . 2006. “Microsatellites for Ecologists: A Practical Guide to Using and Evaluating Microsatellite Markers.” Ecology Letters 9, no. 5: 615–629. 10.1111/j.1461-0248.2006.00889.x. 16643306

[ece372688-bib-0053] Sethuraman, A. , N. M. Gonzalez , C. E. Grenier , et al. 2019. “Continued Misuse of Multiple Testing Correction Methods in Population Genetics‐A Wake‐Up Call?” Molecular Ecology Resources 19, no. 1: 23–26. 10.1111/1755-0998.12969. 30701708

[ece372688-bib-0054] Shafer, A. B. A. , C. R. Peart , S. Tusso , et al. 2017. “Bioinformatic Processing of RAD‐Seq Data Dramatically Impacts Downstream Population Genetic Inference.” Methods in Ecology and Evolution 8, no. 8: 907–917. 10.1111/2041-210x.12700.

[ece372688-bib-0055] Wahlund, S. 1928. “Zusammensetzung Von Population Und Korrelationserscheinung Vom Standpunkt Der Vererbungslehre Aus Betrachtet.” Hereditas 11: 65–106. 10.1111/j.1601-5223.1928.tb02483.x.

[ece372688-bib-0056] Wariss, H. M. , T. X. Liu , H. X. Zhang , J. J. Wu , Z. P. Yang , and W. J. Li . 2025. “Genetic Diversity and Population Structure of the Endangered Medicinal Plant.” Global Ecology and Conservation 58: e03437. 10.1016/j.gecco.2025.e03437.

[ece372688-bib-0057] Weinberg, W. 1908. “On the Demonstration of Heredity in Man.” In Papers on Human Genetics (1963), edited by S. H. Boyer . Prentice Hall.

[ece372688-bib-0058] Yang, Y. Z. , P. W. Sun , C. Y. Ke , et al. 2025. “Towards Climate‐Resilient Conservation: Integrating Genetics and Environmental Factors in Determining Adaptive Units of a Xeric Shrub.” Global Ecology and Conservation 57: e03417. 10.1016/j.gecco.2025.e03417.

