# Supplementary material for: Hardy–Weinberg Equilibrium Filtering in Population Genomics: Empirical Review and Decision Framework for Improved Practice
Source: Ecol Evol. 2026 Jan 9;16(1):e72688. doi: 10.1002/ece3.72688 (PMC12789822; doi:10.1002/ece3.72688)
Supplement: Supplementary file 1 — Table S1: This table summarises the 50 studies in our analysis after screening articles citing Pearman et al. 2022. The 20 additional entries were excluded due to article type, duplication, or inaccessibility (see Section 4 for details). [file ECE3-16-e72688-s001.pdf]

Table S1. This table summarises the 50 studies in our analysis after screening articles citing Pearman et al., 2022. The 20 additional entries were excluded due to article type, duplication, or inaccessibility (see section 4 for details).

No.	Reference	Sequencing approach	HWE analysis	Testing/filtering scheme	Threshold value and correction	Transparency level	Notes
1	Aleman, Dorken, et al. (2024)	lcWGS	No Test	-	-	Explicitly omitted	Explicitly omitted HWE because equilibrium was not expected given broad geographic sampling across species
2	Babaei et al. (2025)	ddRADseq	No Test	-	-	Explicitly omitted	Explicitly omitted HWE filtering
3	Banks, Kirke, Alves, Johnson, and Crook (2024)	DArTseq	No Test	-	-	Explicitly omitted	An university digital recorded report, not peer reviewed; Explicitly omitted HWE filtering.
4	Dimond, Crim, Unsell, Barry, and Toft (2022)	RADseq	No Test	-	-	Explicitly omitted	Explicitly omitted HWE filtering
5	Ellis et al. (2023)	RADseq	No Test	-	-	Explicitly omitted	Explicitly omitted HWE filtering
6	Ellis et al. (2024)	RADseq	No Test	-	-	Explicitly omitted	Explicitly omitted HWE filtering
7	Gautschi et al. (2024)	DArTseq	No Test	-	-	Explicitly omitted	Explicitly omitted HWE filtering

No.	Reference	Sequencing approach	HWE analysis	Testing/filtering scheme	Threshold value and correction	Transparency level	Notes
8	Grant et al. (2022)	RADseq, mtDNA	No Test	-	-	Explicitly omitted	Geographically structured sampling with presence of putative hybrids, so explcitly omitted HWE filtering.
9	Muharromah, Carvajal, Regilme, and Watanabe (2024)	ddRADseq	No Test	-	-	Explicitly omitted	Explicitly omitted HWE filtering
10	K. Phillips (2023)	ddRADseq	No Test	-	-	Explicitly omitted	Explicitly omitted HWE because equilibrium was not expected given mixed-aged or mixed-origin sampling
11	Solmundson et al. (2023)	DArTseq	No Test	-	-	Explicitly omitted	Explicitly omitted HWE filtering
12	Bernal-Durán et al. (2024)	GBS	Filter Applied	Out All	False Discovery Rate (FDR) q = 0.05 (after 10 000 permutations)	High	Out All (half of the analysed populations)
13	González-Salinas, Broitman, Haye,	GBS	Filter Applied	Out All	False Discovery Rate (FDR) q = 0.05	High	Out All (3 of 4 sites)

No.	Reference	Sequencing approach	HWE analysis	Testing/filtering scheme	Threshold value and correction	Transparency level	Notes
	and Segovia (2025)						
14	X. N. Lin et al. (2024)	WGS	Filter Applied	Out All	$p < 0.005$	High	
15	Sainz-Escudero, Vila, Perea, and García-París (2023)	MobiSeq (TE-anchored reduced-representation sequencing)	Filter Applied	Out All	$p < 0.05$	High	
16	Ewart et al. (2023)	DArTseq, mtDNA	Filter Applied	Out All	$p < 0.05$, 1000 permutations	High	
17	Ortiz, Boardman, Ruesink, and Naish (2025)	RADseq	Filter Applied	Out All	Significant FIS ($p < 0.05$)	High	
18	Atsawawaranunt et al. (2023)	DArTseq	Filter Applied	Out Any	$p < 0.01$ (no multiple-testing correction)	High	Intentionaly no filtering before strucure analysis, Out Any for further analysis
19	Byerly et al. (2022)	UCE Capture	Filter Applied	Out Any	Sequential Bonferroni $\alpha = 0.05$	High	

No.	Reference	Sequencing approach	HWE analysis	Testing/filtering scheme	Threshold value and correction	Transparency level	Notes
20	Brittain et al. (2025)	ddRADseq, mtDNA haplotype	Filter Applied	Out Combo	Adjusted p values (multiple-comparison correction)	High	Preprint without peer review yet
21	Markova et al. (2023)	WGS	Filter Applied	Out Within	$p < 0.001$	High	
22	Wolfson (2024)	ddRADseq	Filter Applied	Out Within	$p < 0.05$ (Bonferroni corrected)	High	
23	Gossé, Gonedelé-Bi, Dufour, Danquah, and Gaubert (2024)	Microsatellites, mtDNA	Test Only	Out Combo	$p < 0.003$ (Bonferroni correction)	High	HWE tested across pooled subset (24 individuals, single population, Out Combo)
24	Vandergast et al. (2024)	ddRADseq	Test Only	Out Within	$\alpha = 0.05$ (Benjamini–Yekutieli correction)	High	

No.	Reference	Sequencing approach	HWE analysis	Testing/filtering scheme	Threshold value and correction	Transparency level	Notes
25	Roden et al. (2023)	Microsatellites	Test Only	Out Within	α = 0.05 (Bonferroni correction)	High	
26	Hopken et al. (2023)	RADseq, microhaplotype	Test Only	Out Within	α = 0.05 (Bonferroni correction, 1000 iterations)	High	HWE tests performed on full dataset and within clusters to evaluate Wahlund effect; no HWE filtering was applied.
27	Carvey et al. (2024)	ddRADseq	Filter Applied	Out Any	Not Reported	Moderate	Applied HWE filtering but did not identify any significant deviation
28	Mar-Silva et al. (2024)	RADseq	Filter Applied	Out Combo	Not Reported	Moderate	Pooled within age groups; Reported minimal differences with and without removing HWE deviated loci
29	Dalapicolla et al. (2024)	WGS	Filter Applied	Out All	Not Reported	Moderate	Neutral markers required
30	Lott et al. (2024)	DArTseq	Filter Applied	Out All	Not Reported	Moderate	
31	Segovia, Coral-Santacruz, and Haye (2024)	GBS	Filter Applied	Out All	Not Reported	Moderate	Out all (60% of the analysed populations)

No.	Reference	Sequencing approach	HWE analysis	Testing/filtering scheme	Threshold value and correction	Transparency level	Notes
32	Ryan, Clarke, Piper, Fuller, and Prentis (2025)	DArTseq	Filter Applied	Out Within	Not Reported	Moderate	
33	Holt, Lerma, Raszick, and Medina (2023)	ddRADseq	Filter Applied	Unclear	Heterozogosity > 0.7	Moderate	Preprint without peer review yet; Likely to be Out Within; Citing Pearman et al., 2022, for LD instead of HWE.
34	De Silva (2023)	ddGBS	Filter Applied	Unclear	Heterozogosity > 0.5–0.6	Moderate	He-based filtering applied to remove paralogs or genotyping artefacts; likely Out Combo.
35	de Greef et al. (2022)	WGS	Filter Applied	Unclear	Heterozogosity >0.6	Moderate	Applied HWE filter across all locations; likely pooled filtering. Reported minimal differences with/without HWE removal.
36	Mann (2024)	WGS	Filter Applied	Unclear	p < 0.05 (general), extreme p < 1×10???? flagged	Moderate	No mention of population-level tests, so likely Out Combo.
37	Judson, Hoekstra, and Janzen (2024)	RADseq	Filter Applied	Unclear	p < 10??	Moderate	Possibly within pre-grouped populations or pooled data; implementation unclear

No.	Reference	Sequencing approach	HWE analysis	Testing/filtering scheme	Threshold value and correction	Transparency level	Notes
38	Leveque et al. (2025)	ddRADseq, Allegro Targeted Genotyping	Filter Applied	Unclear	Retaining when ?0.2 < FIS < 0.2	Moderate	Populations previously shown in HWE (microsatellite data); applied FIS-based filtering, unclear test unit.
39	Keller et al. (2023))	WGS	Filter Applied	Unclear	Retaining when ?0.5 < FIS < 0.5	Moderate	Unclear testing unit (population vs. global) for heterozygosity-based HWE filtering.
40	Hauser, Robinson, and Latch (2024)	BestRAD	Test Only	Compare No filtering, Out All and Out Any	Not Reported	Moderate	Explored several HWE filtering approaches (Pearman et al. 2022); "No Filtering" performed best and was used for final analyses.
41	P.-C. Lin et al. (2025)	RADseq	Test Only	Compare No filtering, Out All and Out Any	Not Reported	Moderate	Previously included as a preprint; accepted in October 2025; updated HWE filtering description (Explored several HWE filtering approaches (Pearman et al. 2022); "No Filtering" performed best and was used for final analyses).
42	Myers (2023)	ddRADseq	Test Only	Out Combo	Not Reported	Moderate	Applied HWE evaluation but not for filtering

No.	Reference	Sequencing approach	HWE analysis	Testing/filtering scheme	Threshold value and correction	Transparency level	Notes
43	Cardenas, Mularo, Chavez, and Adams (2023)	ddRADseq	Filter Applied	Unclear	Not Reported	Low	Analyses conducted with both filtered and unfiltered SNPs, but did not provide filtering threshold or compare the results
44	A. Phillips (2024)	WGS	Filter Applied	Unclear	Not Reported	Low	applied with caution, but did not describe in details
45	Gruenthal, Kroska, Wolf, Harris, and Booz (2023)	RADseq	Test Only	Unclear	Not Reported	Low	p-value mentioned for HWE test but not reported. Likely Out Combo or Out All. Explicitly omitted HWE filtering.
46	Moravciková et al. (2023)	GBS	Test Only	Unclear	Not Reported	Low	Applied Heterozygosity-based HWE tests
47	Aleman, Arteaga, Gasca-Pineda, and Bello-Bedoy (2024)	nextRAD	Unclear	-	Not Reported	Opaque	Citing Pearman et al., 2022, for LD instead of HWE.
48	Johnson, Zipfel, Smith, and Welsh (2025)	ddRADseq	Unclear	Out Within	Not Reported	Opaque	In the Introduction seems to support filtering, but in the Methods and Results,

No.	Reference	Sequencing approach	HWE analysis	Testing/filtering scheme	Threshold value and correction	Transparency level	Notes
							no sepcify for whether HWE deviated loci were filtered or not.
49	Stringer et al. (2024)	DArTseq	Unclear	Unclear	Not Reported	Opaque	HWE not mentioned; aiming on examining changes in genetic diversity within species over time
50	Vandervelde (2022)	RADseq	Unclear	Unclear	Not Reported	Opaque	Citing Pearman et al., 2022 for grouping and sequencing errors; HWE Not mentioned

*Abbreviations for methods not mentioned in the main text: FDR – False Discovery Rate; GBS – Genotyping-by-Sequencing; WGS – whole-genome sequencing.

References

Aleman, A., Arteaga, M. C., Gasca-Pineda, J., & Bello-Bedoy, R. (2024). Divergent lineages in a young species: The case of datilillo (*Yucca valida*), a broadly distributed plant from the Baja California Peninsula. *American Journal of Botany, 111*(9), e16385. doi:10.1002/ajb2.16385

Aleman, A., Dorken, M. E., Shafer, A. B. A., Patel, T., Volkova, P. A., & Freeland, J. R. (2024). Development of genomic resources for cattails (*Typha*), a globally important macrophyte genus. *Freshwater Biology, 69*(1), 74–83. doi:10.1111/fwb.14194

Atsawawaranunt, K., Ewart, K. M., Major, R. E., Johnson, R. N., Santure, A. W., & Whibley, A. (2023). Tracing the introduction of the invasive common myna using population genomics. *Heredity (Edinb), 131*(1), 56–67. doi:10.1038/s41437-023-00621-w

Babaei, S., Varkey, D. A., Adamack, A. T., Leblanc, N. M., Puncher, G. N., Parent, G. J., . . . Pavey, S. A. (2025). Genome-wide SNPs reveal novel genetic relationships among Atlantic cod (*Gadus morhua*) from the south coast of Newfoundland, Canada (subdivision 3Ps), Northern cod stock complex, and Gulf of St Lawrence. *PLoS ONE, 20*(3). doi:10.1371/journal.pone.0317768

Banks, S., Kirke, A. K., Alves, F., Johnson, G., & Crook, D. (2024). *Identifying population connectivity of shark bycatch species in Northern Territory waters*. Retrieved from Charles Darwin University: https://www.frdc.com.au/sites/default/files/products/2020-036-DLD.pdf

Bernal-Durán, V., Donoso, D., Pinones, A., Jonsson, P. R., Benestan, L., Landaeta, M. F., . . . Segovia, N. I. (2024). Combining population genomics and biophysical modelling to assess connectivity patterns in an Antarctic fish. *Molecular Ecology, 33*(11), e17360. doi:10.1111/mec.17360

Brittain, K., Muhlbach, T., Cao, R., Mijangos, J. L., Yibarbuk, D., Somaweera, R., . . . Gongora, J. (2025). Unique Population or Unique Species? Genetic Insights into the Pygmy Freshwater Crocodiles of Northern Australia. Retrieved from doi:10.22541/au.174307689.98463113/v1

Byerly, P. A., Chesser, R. T., Fleischer, R. C., McInerney, N., Przelomska, N. A. S., & Leberg, P. L. (2022). Museum Genomics Provide Evidence for Persistent Genetic Differentiation in a Threatened Seabird Species in the Western Atlantic. *Integrative and Comparative Biology, 62*(6), 1838–1848. doi:10.1093/icb/icac107

Cardenas, C. R., Mularo, A. J., Chavez, A. S., & Adams, R. M. M. (2023). Limited genetic differentiation of *Mycetomoellerius mikromelanos* in Parque National Soberanía, Panama: Implications for queen dispersal. *Biotropica, 55*(1), 145–159. doi:10.1111/btp.13171

Carvey, Q. B., Pavey, S. A., Diamond, A. W., Davoren, G. K., Lavoie, R. A., Leblanc, N. M., . . . Major, H. L. (2024). Genetic structure of Atlantic Puffins (*Fratercula arctica*) breeding in Atlantic Canada. *Conservation Genetics, 25*(6), 1159–1174. doi:10.1007/s10592-024-01629-3

Dalapicolla, J., Weir, J. T., Vilaça, S. T., Quaresma, T. F., Schneider, M. P. C., Vasconcelos, A. T. R., & Aleixo, A. (2024). Whole genomes show contrasting trends of population size changes and genomic diversity for an Amazonian endemic passerine over the late quaternary. *Ecology and Evolution, 14*(4), e11250. doi:10.1002/ece3.11250

de Greef, E., Einfeldt, A. L., Miller, P. J. O., Ferguson, S. H., Garroway, C. J., Lefort, K. J., . . . Feyrer, L. J. (2022). Genomics reveal population structure, evolutionary history, and signatures of selection in the northern bottlenose whale, *Hyperoodon ampullatus*. *Molecular Ecology, 31*(19), 4919–4931. doi:10.1111/mec.16643

De Silva, N. P. (2023). *Using ecological and genomic approaches to restore Australian grasslands in the face of global change.* (Doctor of Philosophy (Ph.D.)), Monash University,

Dimond, J. L., Crim, R. N., Unsell, E., Barry, V., & Toft, J. E. (2022). Population genomics of the basket cockle *Clinocardium nuttallii* in the southern Salish Sea: Assessing genetic risks of stock enhancement for a culturally important marine bivalve. *Evolutionary Applications, 15*(3), 459–470. doi:10.1111/eva.13359

Ellis, C. D., MacLeod, K. L., Jenkins, T. L., Rato, L. D., Jézéquel, Y., Pavicic, M., . . . Stevens, J. R. (2023). Shared and distinct patterns of genetic structure in two sympatric large decapods. *Journal of Biogeography, 50*(7), 1271–1284. doi:10.1111/jbi.14623

Ellis, C. D., Paris, J. R., Jenkins, T. L., van Stralen, M. R., Steins, N. A., Schotanus, J., & Stevens, J. R. (2024). Genetic divergence and adaptation of an isolated European lobster population in the Netherlands. *Ices Journal of Marine Science, 81*(6), 1039–1052. doi:10.1093/icesjms/fsae059

Ewart, K. M., Kovacs, T. G. L., Walker, J., Tatarnic, N. J., Clark, H., & Lo, N. T. (2023). Considerable gene flow in troglomorphic cockroach species across a vast subterranean landscape. *Journal of Biogeography, 50*(11), 1967–1980. doi:10.1111/jbi.14707

Gautschi, D., Heinsohn, R., Ortiz-Catedral, L., Stojanovic, D., Wilson, M., Crates, R., . . . Neaves, L. (2024). Genetic diversity and inbreeding in an endangered island-dwelling parrot population following repeated population bottlenecks. *Conservation Genetics, 25*(3), 725–737. doi:10.1007/s10592-023-01599-y

González-Salinas, C., Broitman, B. R., Haye, P. A., & Segovia, N. I. (2025). First evidence of fine-scale adaptive genetic structure in farmed

populations of *Mytilus* mussels. *Aquaculture, 609*. doi:10.1016/j.aquaculture.2025.742817

Gossé, K. J., Gonedelé-Bi, S., Dufour, S., Danquah, E., & Gaubert, P. (2024). Conservation genetics of the white-bellied pangolin in West Africa: A story of lineage admixture, declining demography, and wide sourcing by urban bushmeat markets. *Ecology and Evolution, 14*(3), e11031. doi:10.1002/ece3.11031

Grant, E. H. C., Mulder, K. P., Brand, A. B., Chambers, D. B., Wynn, A. H., Capshaw, G., . . . Bell, R. C. (2022). Speciation with gene flow in a narrow endemic West Virginia cave salamander (*Gyrinophilus subterraneus*). *Conservation Genetics, 23*(4), 727–744. doi:10.1007/s10592-022-01445-7

Gruenthal, K. M., Kroska, A. C., Wolf, N., Harris, B. P., & Booz, M. D. (2023). High Spatiotemporal Genetic Connectivity in the Pacific Razor Clam (*Siliqua patula*) throughout Cook Inlet, Alaska. *Journal of Shellfish Research, 42*(3), 371–380. doi:10.2983/035.042.0304

Hauser, S., Robinson, S., & Latch, E. (2024). Genomic analysis of population history for Hawaiian monk seals. *Endangered Species Research, 53*, 327–340. doi:10.3354/esr01308

Holt, J. R., Lerma, J. M., Raszick, T. J., & Medina, R. F. (2023). High-Resolution Population Genetic Structure of Tawny Crazy Ant (*Nylanderia fulva Mayr*: Hymenoptera: Formicidae) from the Origin in South America and Introduced Regions of the United States. Retrieved from doi:10.21203/rs.3.rs-2399319/v1

Hopken, M. W., Piaggio, A. J., Abdo, Z., Chipman, R. B., Mankowski, C. P., Nelson, K. M., . . . Gilbert, A. T. (2023). Are rabid raccoons (*Procyon lotor*) ready for the rapture? Determining the geographic origin of rabies virus-infected raccoons using RADcapture and microhaplotypes. *Evolutionary Applications, 16*(12), 1937–1955. doi:10.1111/eva.13613

Johnson, A., Zipfel, K., Smith, D., & Welsh, A. (2025). Conservation Genomics of West Virginia Walleye (*Sander vitreus*): Impact of Minor Allele Frequency Thresholds on Population Structure and Potential Adaptive Divergence Inferences. *DNA, 5*(1), 14. doi:10.3390/dna5010014

Judson, J. M., Hoekstra, L. A., & Janzen, F. J. (2024). Demographic history and genomic signatures of selection in a widespread vertebrate ectotherm. *Molecular Ecology, 33*(5), e17269. doi:10.1111/mec.17269

Keller, A. G., Dahlhoff, E. P., Bracewell, R., Chatla, K., Bachtrog, D., Rank, N. E., & Williams, C. M. (2023). Multi-locus genomic signatures of local adaptation to snow across the landscape in California populations of a willow leaf beetle. *Proceedings of the Royal Society B-Biological*

Sciences, 290(2005), 20230630. doi:10.1098/rspb.2023.0630

Leveque, A., Arnaud, J. F., Vignon, V., Mazoyer, C., Gode, C., & Duputie, A. (2025). Development of a panel of SNP loci in the emblematic southern damselfly (*Coenagrion mercuriale*) using a hybrid method: pitfalls and recommendations for large-scale SNP genotyping in a nonmodel endangered species. *Journal of Heredity, 116*(3), 255–271. doi:10.1093/jhered/esae073

Lin, P.-C., Wu, C.-W., Lee, C.-R., Huang, J.-P., Lin, C.-P., Wang, L.-J., & Hsu, Y. H. (2025). Detecting local adaptation under weak genetic structure in an endemic damselfly: an integrative eco-evolutionary approach. *BMC Ecology and Evolution, In press*. doi:10.1186/s12862-025-02462-z

Lin, X. N., Ma, C. Y., Hu, L. S., Liao, M. L., Ma, L. X., Teske, P. R., . . . Dong, Y. W. (2024). Genomics-Informed Range Predictions Under Global Warming Reveal Reduced Adaptive Diversity Whilst Buffering Range Shifts for a Marine Snail. *Global Change Biology, 30*(11), e17571. doi:10.1111/gcb.17571

Lott, M. J., Frankham, G. J., Eldridge, M. D. B., Alquezar-Planas, D. E., Donnelly, L., Zenger, K. R., . . . Neaves, L. E. (2024). Reversing the decline of threatened koala (*Phascolarctos cinereus*) populations in New South Wales: Using genomics to enhance conservation outcomes. *Ecology and Evolution, 14*(8), e11700. doi:10.1002/ece3.11700

Mann, M. A. (2024). *Deciphering the Molecular Biology of Diaphorina citri and "Candidatus Liberibacter asiaticus" Interactions: Insights Into Huanglongbing and Vector Capacity.* (Partial Fulfillment of the Requirements for the Degree of Doctor of Philosophy), Cornell University,

Mar-Silva, A. F., Díaz-Jaimes, P., Ochoa-Zavala, M., Ortega-Garcia, S., Sotil, G., Alegre, A., . . . Carvajal-Rodríguez, J. M. (2024). A genomic approach for the identification of population management units for the dolphinfish (*Coryphaena hippurus*) in the eastern Pacific. *Frontiers in Marine Science, 10*. doi:10.3389/fmars.2023.1294509

Markova, S., Lanier, H. C., Escalante, M. A., da Cruz, M. O. R., Hornikova, M., Konczal, M., . . . Kotlik, P. (2023). Local adaptation and future climate vulnerability in a wild rodent. *Nature Communications, 14*(1), 7840. doi:10.1038/s41467-023-43383-z

Moravciková, N., Kasarda, R., Zidek, R., McEwan, J. C., Brauning, R., Landete-Castillejos, T., . . . Pokorádi, J. (2023). Traces of Human-Mediated Selection in the Gene Pool of Red Deer Populations. *Animals, 13*(15). doi:10.3390/ani13152525

Muharromah, A. F., Carvajal, T. M., Regilme, M. A. F., & Watanabe, K. (2024). Fine-scale adaptive divergence and population genetic structure of *Aedes aegypti* in Metropolitan Manila. *Parasites & Vectors, 17*(1). doi:10.1186/s13071-024-06300-x

Myers, N. A. (2023). *The Landscape Genetics of Novel Environments: How Urban Environments Structure the Introduced Lizards of Honolulu*. (Master of Science), University of Hawai'i, Hawai'i, USA. Retrieved from https://hdl.handle.net/10125/107933

Ortiz, B. A. B., Boardman, F. C., Ruesink, J. L., & Naish, K. A. (2025). Adaptive Genetic Differentiation Between Spatially Proximate Annual and Perennial Life History Types of a Marine Foundation Species. *Molecular Ecology, 34*(8), e17730. doi:10.1111/mec.17730

Phillips, A. (2024). *Polyploidy in Andropogon gerardi: A Series of Happy Accidents*. (Doctor of Philosophy), University of California, Davis, California, USA. Retrieved from https://escholarship.org/uc/item/7fb646v4

Phillips, K. (2023). *Juvenile Dispersal and Genetic Connectivity in the Sea Turtle 'Lost Years'*. (Doctor of Philosophy (Ph.D.) Electronic Theses and Dissertations), University of Central Florida, Retrieved from https://purls.library.ucf.edu/go/DP0027122

Roden, S. E., Horne, J. B., Jensen, M. P., FitzSimmons, N. N., Balazs, G. H., Farman, R., . . . Dutton, P. H. (2023). Population structure of Pacific green turtles: a new perspective from microsatellite DNA variation. *Frontiers in Marine Science, 10*, 10:1116941. doi:10.3389/fmars.2023.1116941

Ryan, J. L., Clarke, A. R., Piper, A. M., Fuller, S., & Prentis, P. J. (2025). Gene Flow and Abundance of a Tropical Fruit Fly in a Horticultural Landscape Mosaic in Eastern Australia Is Limited by Cleared Grazing Land and Area-Wide Management. *Evolutionary Applications, 18*(4), e70097. doi:10.1111/eva.70097

Sainz-Escudero, L., Vila, M., Perea, S., & García-París, M. (2023). Large effective size as determinant of population persistence in Anostraca (Crustacea: Branchiopoda). *Conservation Genetics, 24*(6), 675–692. doi:10.1007/s10592-023-01534-1

Segovia, N. I., Coral-Santacruz, D., & Haye, P. A. (2024). Genetic homogeneity and weak signatures of local adaptation in the marine mussel *Mytilus chilensis*. *Scientific Reports, 14*(1), 21081. doi:10.1038/s41598-024-71944-9

Solmundson, K., Bowman, J., Manseau, M., Taylor, R. S., Keobouasone, S., & Wilson, P. J. (2023). Genomic population structure and inbreeding history of Lake Superior caribou. *Ecology and Evoution, 13*(7), e10278. doi:10.1002/ece3.10278

Stringer, E. J., Gruber, B., Sarre, S. D., Wardle, G. M., Edwards, S. V., Dickman, C. R., . . . Duncan, R. P. (2024). Boom-bust population dynamics drive rapid genetic change. *Proceedings of the National Academy of Sciences of the United States of America, 121*(18), e2320590121. doi:10.1073/pnas.2320590121

Vandergast, A. G., Kus, B. E., Wood, D. A., Mitelberg, A., Smith, J. G., & Milano, E. R. (2024). High inter-population connectivity and occasional gene

flow between subspecies improves recovery potential for the endangered Least Bell's Vireo. *Ornithological Applications, 126*(3), duae009. doi:10.1093/ornithapp/duae009

Vandervelde, C. (2022). *Population structure of bigmouth buffalo (Ictiobus cyprinellus) across Canada and the United States.* (Master of Science Electronic Theses), University of Manitoba Canada. Retrieved from http://hdl.handle.net/1993/37122

Wolfson, D. (2024). *A Multi-Faceted Evaluation of a Reintroduced Waterfowl Species: Migration Ecology, Ecotoxicology, and Population Genetics of Trumpeter Swans in the Midwest.* (Doctor of Philosophy), University of Minnesota Twin Cities, Minnesota, USA. Retrieved from https://hdl.handle.net/11299/269249
